# Methylation mediated by an anthocyanin, *O*-methyltransferase, is involved in purple flower coloration in *Paeonia*

**DOI:** 10.1093/jxb/erv365

**Published:** 2015-07-23

**Authors:** Hui Du, Jie Wu, Kui-Xian Ji, Qing-Yin Zeng, Mohammad-Wadud Bhuiya, Shang Su, Qing-Yan Shu, Hong-Xu Ren, Zheng-An Liu, Liang-Sheng Wang

**Affiliations:** ^1^Key Laboratory of Plant Resources/ Beijing Botanical Garden, Institute of Botany, Chinese Academy of Sciences, Beijing 100093, PR China; ^2^University of Chinese Academy of Sciences, Beijing 100049, PR China; ^3^State Key Laboratory of Systematic and Evolutionary Botany, Institute of Botany, Chinese Academy of Sciences, Beijing 100093, PR China; ^4^MOgene Green Chemicals, Saint Louis, MO 63132, USA

**Keywords:** Anthocyanin *O*-methyltransferase, catalytic activity, flavonoids, flower coloration, *Paeonia*, single amino acid substitution.

## Abstract

Anthocyanins are major pigments in plants. Methylation plays a role in the diversity and stability of anthocyanins. However, the contribution of anthocyanin methylation to flower coloration is still unclear. We identified two homologous anthocyanin *O*-methyltransferase (*AOMT*) genes from purple-flowered (*PsAOMT*) and red-flowered (*PtAOMT*) *Paeonia* plants, and we performed functional analyses of the two genes *in vitro* and *in vivo*. The critical amino acids for AOMT catalytic activity were studied by site-directed mutagenesis. We showed that the recombinant proteins, PsAOMT and PtAOMT, had identical substrate preferences towards anthocyanins. The methylation activity of PsAOMT was 60 times higher than that of PtAOMT *in vitro*. Interestingly, this vast difference in catalytic activity appeared to result from a single amino acid residue substitution at position 87 (arginine to leucine). There were significant differences between the 35S::*PsAOMT* transgenic tobacco and control flowers in relation to their chromatic parameters, which further confirmed the function of *PsAOMT in vivo*. The expression levels of the two homologous *AOMT* genes were consistent with anthocyanin accumulation in petals. We conclude that AOMTs are responsible for the methylation of cyanidin glycosides in *Paeonia* plants and play an important role in purple coloration in *Paeonia* spp.

## Highlight

A single amino acid residue substitution at position 87 from arginine to leucine of AOMT is vital for anthocyanin methylation in *Paeonia* plants, especially for purple flower coloration.

## Introduction

The colours of flowers, fruits, and leaves are critically important traits in plants for UV protection and attracting pollinators and seed-dispersing organisms (Grotewold, 2006). The major classes of pigments that cause plant coloration are flavonoids, carotenoids, and betalains. The flavonoids are the best-studied class of pigments and are known to be widely distributed among the angiosperms (Tanaka *et al.*, 2008). Flavonoids play important roles in various ecological and physiological processes in plants, including pigmentation, UV absorption, antioxidation, defence responses, and signal transduction, among others (Tahara, 2007).

**Fig. 1.**
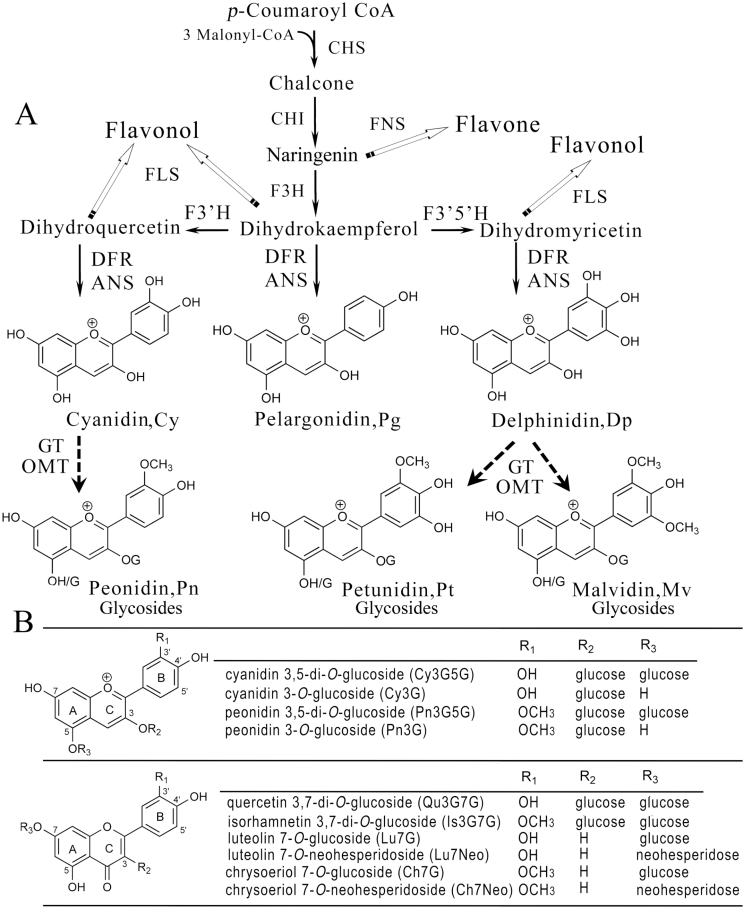
Biochemical pathway and chemical structural information regarding selected flavonoid compounds in plants. (A) Schematic diagram of the biosynthetic pathways of the major flavonoids (Rausher *et al.*, 2008; Wessinger and Rausher, 2012). Dashed arrows represent the unclear steps. The names and structures of three anthocyanidin compounds are indicated. The enzyme names in black boxes are as follows: CHS, chalcone synthase; CHI, chalcone isomerase; F3H, flavanone 3-hydroxylase; F3′H, flavonoid 3′-hydroxylase; F3′5′H, flavonoid 3′,5′-hydroxylase; DFR, dihydroflavonol 4-reductase; ANS, anthocyanidin synthase; FNS, flavones synthase; FLS, flavonol synthase; GT, glycosyltransferase; OMT, *O*-methyltransferase. (B) Chemical structures of anthocyanins, flavones, and flavonols in *Paeonia* flower petals (Wang *et al.*, 2001; Li *et al.*, 2009).

Anthocyanins are the largest group of flavonoids and the greatest contributors to floral coloration, providing the basis for orange, pink, red, magenta, scarlet, purple, blue, and blue/black flower colours (Tanaka *et al.*, 2009).The basic biosynthetic pathways of three major anthocyanidins, namely pelargonidin, cyanidin, and delphinidin, have been well characterized (Fig. 1A). Further modifications to anthocyanidins including methylation, glycosylation, and acylation in these three aglycones produce a wide variety of anthocyanin compounds that strengthen the flower colour phenotypes (Rausher, 2008; Wessinger and Rausher, 2012). Previous studies that investigated the methylation of flavonoids have focused primarily on flavones and isoflavones (Ibrahim *et al.*, 1998; Noel *et al.*, 2003; Lam *et al.*, 2007; Wang, 2011). The methylation of anthocyanins was first reported in petunia at the 3′ and 5′ positions of aglycones (Wiering and Devlaming, 1977; Jonsson *et al.*, 1983; Brugliera *et al.*, 2003; Provenzano *et al.*, 2014). Anthocyanin *O*-methyltransferase (AOMTs) were isolated from different grape cultivars, named VvAOMT and FAOMT, and characterized *in vitro* and *in vivo*, and they were found to be anthocyanin 3′- and 3′,5′-*O*-methyltransferase (Hugueney *et al.*, 2009; Lucker *et al.*, 2010). Akita *et al.* (2011) revealed a gene (*CkmOMT2*) from purple-flowered fragrant cyclamen, and an enzyme assay with heterologously expressed CkmOMT2 *in vitro* demonstrated that CkmOMT2 exhibited methylation activity with anthocyanins. A red/purple flower colour in a fragrant cyclamen mutant was bred by ion-beam irradiation, which was caused by the loss of the *CkmOMT2* region (Kondo *et al.*, 2009). Previous studies on the chromatic properties of anthocyanins from red grapes (Heredia *et al.*, 1998) and tree peony petals (Sakata *et al.*, 1995) demonstrated the effects of methylation on the hue; specifically, the higher the number of methoxyl groups, the more pronounced the shift towards purple. In addition, it has been proposed that the methylation of B-ring hydroxyl groups causes a small shift towards red (Tanaka *et al.*, 2008). Although there has been some progress on anthocyanin methylation, a large portion of methyltransferases of plant origin needs to be further characterized, as several questions remain to be answered, such as the molecular mechanism of anthocyanin methylation, the way in which methyltransferase takes part in the methylation of anthocyanins *in vivo*, and the relationship between anthocyanin methylation and floral coloration.

Methylation is an alkylation reaction that transfers an activated methyl group from *S*-adenosylmethionine (SAM) to the *N*-, *C*-, *O*-, or *S*-nucleophiles of acceptor molecules (Klimasauskas and Weinhold, 2007). Most methyltransferases methylate hydroxyl and carboxyl moieties, and are referred to as *O*-methyltransferases (OMTs). A large family of OMTs in plants contributes to the vast structural and functional diversity of plant natural products (Wang, 2011), which can be classified into two types according to their sequence homology and substrate variance (Joshi and Chiang, 1998; Noel *et al.*, 2003; Lam *et al.*, 2007). Type I OMTs include a group of homodimeric OMTs with molecular weights of 38–43kDa and are cation independent, which methylate both flavonoids and isoflavonoids. Type II OMTs have lower molecular weights (23–27kDa) and are cation dependent. Most type II OMTs have been shown to be specific for caffeoyl coenzyme A esters of phenylpropanoids (CCoAOMTs), which are thought to be key enzymes in the biosynthesis of lignin (Ye *et al.*, 1994). However, Ibdah *et al.* (2003) reported that a novel Mg^2+^-dependent PFOMT from *Mesembryanthemum crystallinum* (ice plant) with a molecular weight of 26.6kDa and high similarity to type II OMTs was specific for the methylation of flavonols and caffeoyl-CoA. Thus, there is a novel subclass within type II OMTs with diverse substrates that are not restricted to lignin synthesis. The identification of cation-dependent OMTs such as ROMT15/17 from *Oryza sativa* (Lee *et al.*, 2008), VvAOMT from *Vitis vinifera* (Hugueney *et al.*, 2009), FAOMT from *V. vinifera* (Lucker *et al.*, 2010), and ObF8OMT (Berim and Gang, 2013) with preferences for flavonoid substrates confirmed the appropriateness of the definition of the new subclass of type II OMTs. This progress highlights the occurrence of novel flavonoid methylation enzymes that differ from type I OMTs and underscores the need to further investigate their catalytic mechanisms.

Plants in the genus *Paeonia* are important ornamentals throughout the world. These beautiful plants have large flowers in a variety of colours and shapes. China has a long history of cultivating and breeding *Paeonia* cultivars and has rich collections of germplasm resources (Ji *et al.*, 2012). However, a large proportion of the available cultivars have a purple or ‘purplish’ flower, but rarely a red flower. Our previous studies on flavonoids from the flowers of various *Paeonia* spp. demonstrated that peonidin derivatives were the major anthocyanidins that accumulated in most cultivars (Wang *et al.*, 2001; Jia *et al.*, 2008; Li, 2010), indicating that methylation modifications of anthocyanins are prevalent in *Paeonia* spp. As such, these plants provide a good model system for the investigation of methylation mechanisms and their influence on flower coloration.

In this study, we characterized an AOMT (PsAOMT) from a purple-flowered plant from the genus *Paeonia* and characterized its homologue PtAOMT from another plant in the genus *Paeonia* with a vivid red flower using both *in vitro* and *in vivo* methods. The catalytic activity PtAOMT was 60-fold less than that of PsAOMT. By using site-directed mutagenesis, we demonstrated that the vast difference in catalytic activities between these two enzymes was caused by the substitution of one key amino acid. This work characterized the subclass of type II OMTs by integrating biochemical, molecular, and phytochemical analysis, which will support an understanding of the anthocyanin methylating mechanism and shed light on its influence on flower coloration. The efficient enzyme PsAOMT and its key amino acid are responsible for effective activity and could be applied to the specifically targeted molecular breeding of ornamental and crop plants or the development of healthy and beneficial products.

## Materials and methods

### Chemical sources

Cyanidin, delphinidin, peonidin, pelargonidin 3-*O*-glucoside (Pg3G), and delphinidin 3-*O*-glucoside (Dp3G) were purchased from Extrasynthese (Genay, France). Quercetin, quercetin 3-*O*-rutinoside (Qu3R), and caffeic acid were obtained from the National Institute for the Control of Pharmaceutical and Biological Products (Beijing, China). Cyanidin 3,5-di-*O*-glucoside (Cy3G5G), cyanidin 3-*O*-glucoside (Cy3G), kaempferol 3-*O*-glucoside (Km3G), kaempferol, luteolin, apigenin, naringenin, and epicatechin were purchased from Sigma-Aldrich (Shanghai, China). Analytical-grade methanol and acetonitrile were obtained from Promptar (Elk Grove, CA, USA).

### Plant materials and culture conditions

A tree peony cultivar (*Paeonia suffruticosa* cv. ‘Gunpohden’) and an herbaceous peony *Paeonia tenuifolia* were used. The plants were grown at the Beijing Botanical Garden. The tobacco and strawberry plants were cultivated in a greenhouse under a 14h light/10h dark photoperiod. The temperature was maintained at 25 °C during the light period and 18 °C during the dark period.

### Cloning candidate *AOMT* cDNA and phylogenetic analysis

An open reading frame (ORF) of a segment of expressed sequence tag (FE529149) from a cDNA library of the tree peony (Shu *et al.*, 2009) is highly homologous to genes for the flavonoid OMT (PFOMT) from *M. crystallinum* (Ibdah *et al.*, 2003) and anthocyanin OMT (VvAOMT) from grapevine (Hugueney *et al.*, 2009) and was used for a reference sequence for cloning *AOMT*s from *Paeonia* plants. Total RNA was isolated from the two *Paeonia* petals with an RNAprep pure kit (Tiangen, Beijing, China). One microgram of total RNA was used as the template for cDNA synthesis with Moloney murine leukemia virus reverse transcriptase (Promega, WI, USA). The ORFs of *PsAOMT* and *PtAOMT* were cloned with high-fidelity PrimerSTAR HS polymerase (TaKaRa, Ohtsu, Japan) by using the AOMT forward/reverse primers (Supplementary Table S1, available at *JXB* online) from *P. suffruticosa* cv. ‘Gunpohden’ and *P. tenuifolia*. The amino acid sequences of PsAOMT and PtAOMT were aligned with CLUSTAL X (Thompson *et al.*, 1997) and refined manually. MEGA 5.1 software was used to reconstruct a phylogenetic tree by using the maximum-likelihood test method (Tamura *et al.*, 2011), with 1000 bootstrap replicates.

### Heterologous expression of AOMTs and site-directed mutagenesis

The sequenced cDNA of *AOMT* was inserted into the pMAL-c5X expression vector (NEB, MA, USA), which contains a maltose-binding protein tag. Recombinant AOMTs were purified with an amylose resin column (NEB). Site-directed mutagenesis was performed by using a Fast Mutagenesis System kit (TransGen, Beijing, China). The sequences of the primers used for this protocol are given in Supplementary Table S1.

### Characterizing the recombinant AOMTs

The assay reaction conditions were optimized prior to performing quantitative analyses. The influence of pH on AOMT activity was assessed within a pH range of 4.5–8.5 using MES (pH 4.5–6.5) and Tris/HCl (pH 7.5–8.5) buffers. The effect of divalent cations on the enzyme activity was estimated by adding aqueous solutions of MgCl_2_, CaCl_2_, ZnCl_2_, MnCl_2_, CoCl_2_, or EDTA (all at 10mM final concentration) to the reaction mixture. The optimal concentrations of metal ions were assessed by testing different concentrations of MgCl_2_ (0.1, 0.2, 0.5, 1.0, 5.0, and 10mM). The optimized conditions were as follows: purified recombinant AOMT (2 μg) was assayed in a final volume of 200 μl containing 200 μM SAM, 1.0mM MgCl_2_, 14mM β-mercaptoethanol, 100mM Tris/HCl (pH 7.5), and 20 μM flavonoid substrates (the chemical structures are shown in Supplementary Fig. S1, available at *JXB* online). Incubation was performed at 35 °C and stopped with 800 μl of methanol containing 2% formic acid, followed by centrifugation at 12 000rpm for 10min. The upper liquid was prepared for high-performance liquid chromatography (HPLC) analysis, and 20 µl of reaction sample was loaded. Anthocyanin and flavonol profiles were recorded at 525 and 350nm, respectively. The substrates and products were identified by comparison with standards and HPLC electrospray ionization mass spectrometry (HPLC-ESI/MS). For the kinetic studies, purified AOMT was incubated under the above optimized conditions with the exception of a range of substrate concentrations from 5 to 200 μM for *K*_m_ determination (Hugueney *et al.*, 2009). Reaction products were analysed by HPLC, using the method reported by Yang *et al.* (2009).

### Molecular modelling of the PsAOMT and PtAOMT active sites

Three-dimensional models of PtAOMT and PsAOMT were generated by using the I-TASSER Protein Structure and Function Predictions web server (Roy *et al.*, 2010). Homology models were built by using the known three-domensional (3D) structure of caffeoyl coenzyme, a 3-*O*-methyltransferase (CCoAOMT, PDB code 1SUI) from alfalfa (Ferrer *et al.*, 2005). The best models of PsAOMT and PtAOMT were evaluated based on their template modelling score (0.90), root mean square deviation (1.75), and sequence identity (54.0%) in relation to the 3D structure of CCoAOMT. Because recombinant AOMTs form dimers in solution, PsAOMT and PtAOMT were modelled as homodimers by using the COOT program (Krissinel and Henrick, 2004). Substrate-binding sites were predicted by docking Cy3G5G with the PsAOMT dimer 3D model in the SWISDOCK program (Grosdidier *et al.*, 2011).

### Transient expression in strawberry fruit

*Fragaria×ananassa* cv. ‘Hongyan’ mature plants with fruits that were just turning red were used for these experiments. A single colony of *Agrobacterium* strain EHA105 with *PsAOMT* or *PtAOMT* and a single colony with *Arabidopsis PRODUCTION OF ANTHOCYANIN PIGMENT1* (*PAP1*; GenBank accession no. AF325123) were cultured and diluted to an OD_600_ of 0.1–0.3, and injected into strawberry fruits according to the method of Hoffmann *et al.* (2006). The infiltrated fruits were harvested after 4 d, and the extracts were analysed according to Yang *et al.* (2009).

### Tobacco transformation

*PsAOMT* and *PtAOMT* were cloned into the *pBI121* binary vector (Clontech). Sequence-confirmed constructs were then introduced into *Agrobacterium* strain EHA105 by electroporation. A single positive colony was co-cultured with leaf sections of sterile *Nicotiana tabacum* cv. Nc89 according to a previously reported protocol (Horsch *et al.*, 1985). Finally, kanamycin-resistant plantlets were transferred to soil mix, acclimatized, and grown in a greenhouse. Positive transgenic lines were selected by PCR, and empty-plasmid transgenic plantlets were used as controls. The transgenic tobacco lines with similar expression levels of *PsAOMT* and *PtAOMT* were used for further study.

### Quantitative PCR (qPCR) analysis of *AOMT* expression

Flower petals at five different developmental phases were sampled in triplicate. Total RNA was prepared as described above. Quantitative assays of gene expression were performed using an UltraSYBR Mixture (CWBIO, Beijing, China) and analysed with a Stratagene (CA, USA) Mx3000P instrument as described by Lan *et al.* (2009). The relative quantification of mRNA transcripts was performed in triplicate with normalization to *Actin* (GenBank accession no. JN105299) (Zhao *et al.*, 2012). The primers used in the qPCR analysis were flanked by an intron (Supplementary Table S1). The PCR products were sequenced to confirm that the correct gene was amplified. AOMT expression in the roots, stems, leaves, and sepals was also studied.

### HPLC diode-array detection (HPLC-DAD) and HPLC-MS analyses of flavonoids extracts

The flavonoids (anthocyanins, flavones, and flavonols) in petals from *P. suffruticosa* cv. ‘Gunpohden’ and *P. tenuifolia* at five stages, in transgenic tobacco flowers, and in strawberry fruits were extracted using a method described by Yang *et al.* (2009). The extraction protocol was as follows: transgenic tissues (tobacco and strawberry) were extracted with 2% formic acid/methanol (v/v) assisted by sonication at 20 °C for 20min. All extracts were filtered through a 0.22 μm membrane before injection. The solvent and gradient method for the separation of transgenic tobacco flower extracts, strawberry fruit extracts, and enzyme assay extracts was as follows: solvent A, 10% aqueous formic acid; solvent B, 0.1% formic acid in acetonitrile; constant gradient from 5 to 40% B within 25min, maintain 40% B for 5min, and then return to 5% B in 5min. The flow rate was 0.8ml min^–1^. The column temperature was maintained at 30 °C, and 10 μl of analyte was injected. DAD data were recorded from 200 to 800nm.

An HPLC-ESI-MS^n^ system was used as described by Zhu *et al.* (2012). The positive-ion mode was adapted for anthocyanins; both the positive-ion and negative-ion modes were employed for colourless flavonoids. MS spectra were recorded over an *m*/*z* range of 100–1000.

The identification of anthocyanins and colourless flavonoids was according to past research on *Paeonia* plants (Wang *et al.*, 2001; Li *et al.*, 2009). The anthocyanins and colourless flavonoids were quantified using Cy3G and Qu3R as standards, respectively, by linear regression. The anthocyanin quantity was expressed as μg of Cy3G equivalents g^–1^ of dry weight (DW), and by using the calibration curve, the following was obtained: anthocyanin (mAU)=0.474 [Cy3G (μg ml^–1^)] – 1.275 (*r* ^2^=0.999). Colourless flavonoids were expressed as μg of Qu3R equivalents g^–1^ of DW, with the following calibration curve: (mAU)=0.437 [Qu3R (μg ml^–1^)] – 2.485 (*r* ^2^=0.997). All samples were analysed in triplicate.

### Colour measurements (CIELab system)

Chromatic analyses were performed based on the CIE 1976 (*L* ^*^ a*b ^*^) system (Gonnet, 1998). The colours were expressed as L*, a*, and b* values. The value of L* represents lightness, from black (0) to white (100); a* describes red (positive) to green (negative); b* describes yellow (positive) to blue (negative); and C* represents the chroma or saturation of the colour. The colour parameters were measured with an NF333 spectrophotometer according to Zhu *et al.* (2012).

## Results

### Flavonoid accumulation during the floral development of *Paeonia* plants

**Fig. 2.**
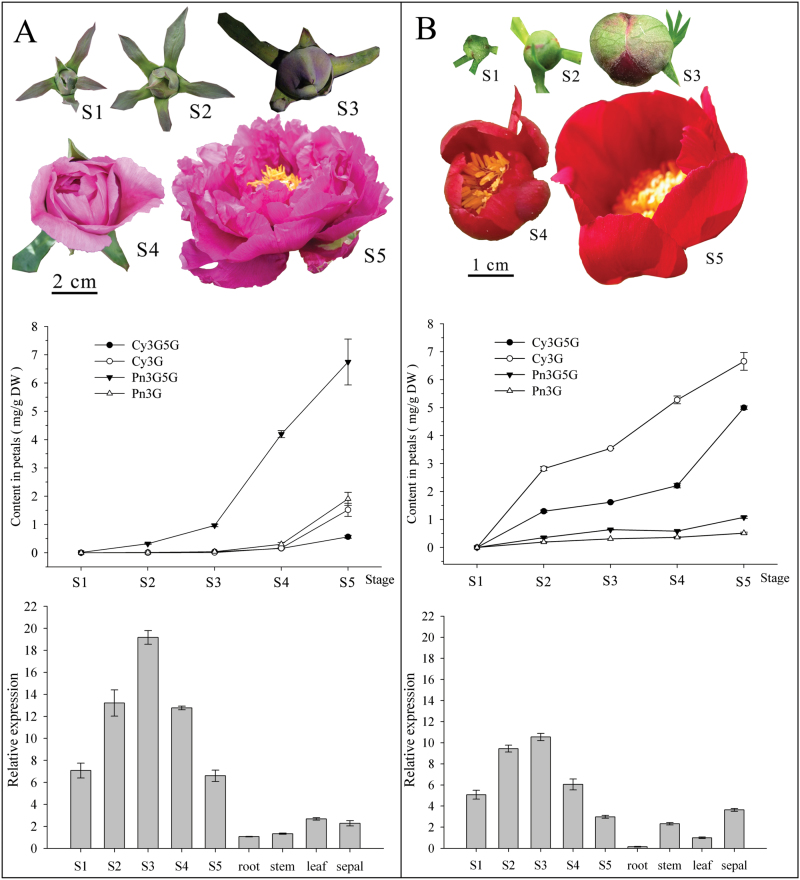
Flowers from five developmental stages and the anthocyanin accumulation at five developmental stages (S1–S5) from *Paeonia suffruticosa* cv. ‘Gunpohden’ (A) and *P. tenuifolia* (B), as well as expression patterns of *AOMT* transcripts at the corresponding developmental stages and in different tissues, measured by qPCR. The expression values have been normalized against the *Actin* gene and are expressed as relative abundances.

We investigated the flavonoid profiles of purple flower petals from *P. suffruticosa* cv. ‘Gunpohden’ and the vivid red flower petals of *P. tenuifolia* at five different developmental stages (S1–S5, from colourless to full floral expansion) (Fig. 2). Petal anthocyanins were identified as Cy3G, Cy3G5G, peonidin 3-*O*-glucoside (Pn3G), and peonidin 3,5-di-*O*-glucoside (Pn3G5G) according to Wang *et al.* (2001) (Fig. 1B). The anthocyanins accumulated during development and reached a maximum at the bloom stage (S5). The total concentration of anthocyanins were 10.73±2.29mg g^–1^ of DW and 13.25±0.76mg g^–1^ of DW at S5 for *P. suffruticosa* cv. ‘Gunpohden’ and *P. tenuifolia*, respectively. Interestingly, peonidin-derived anthocyanins (Pn3G and Pn3G5G, 94%) were the primary anthocyanins of purple flowers (*P. suffruticosa* cv. ‘Gunpohden’), and cyanidin-derived anthocyanins (Cy3G and Cy3G5G, 76%) were the primary anthocyanins in vivid red flowers (*P. tenuifolia*) (Fig. 2 and Supplementary Table S2, available at *JXB* online). Other flavonoid compound profiles of *P. suffruticosa* cv. ‘Gunpohden’ and *P. tenuifolia* flowers were analysed at 350 and 280nm, respectively (Supplementary Fig. S2, available at *JXB* online). Co-pigments (flavonols and flavones) were abundant in the petals of *P. suffruticosa* cv. ‘Gunpohden’, and they were primarily quercetin 3,7-di-*O*-glucoside (Qu3G7G), isorhamnetin 3,7-di-*O*-glucoside (Is3G7G), luteolin 7-*O*-glucoside (Lu7G), chrysoeriol 7-*O*-glucoside (Ch7G), luteolin 7-*O*-neohesperidose (Lu7Neo) and chrysoeriol 7-*O*-neohesperidose (Ch7Neo) according to Li *et al.*(2009) (Fig. 1B). The amounts at different developmental stages are shown in Supplementary Fig. S3, available at *JXB* online. By contrast, the red flowers of *P. tenuifolia* contained negligible amounts of co-pigments (Supplementary Fig. S2). Given the hypothesis that anthocyanins are responsible for coloration, the anthocyanin methyltransferase might be the reason for the observed colour difference given the anthocyanins that were characterized from these two plants.

### *In silico* analysis of candidate *AOMT*s

**Fig. 3.**
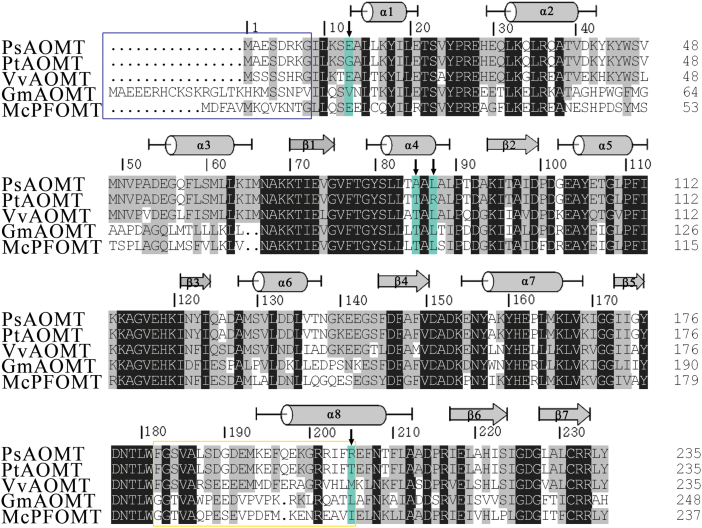
Sequence alignments of PsAOMT and PtAOMT with predicted secondary structural elements. The reference sequences VvAOMT (grapevine, BQ796057), GmAOMT (black soybean, ADX43927) and McPFOMT (ice plant, AY145521) are also included. α-Helices and β-strands are represented as cylinders and arrows, respectively. The residues conserved in all OMTs are shaded. (This figure is available in colour at *JXB* online.)

**Fig. 4.**
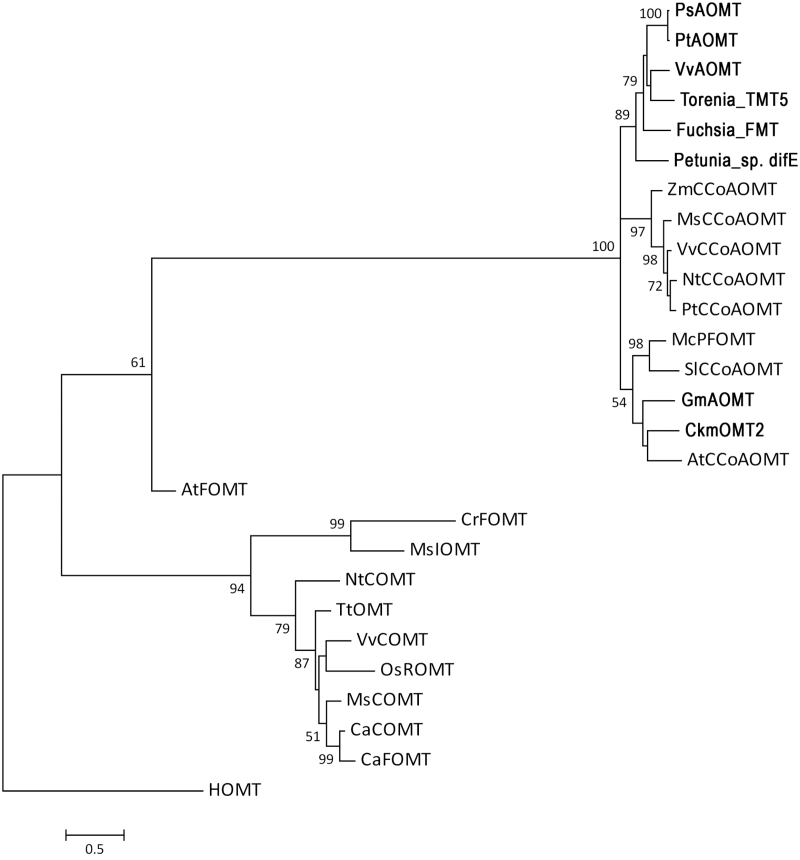
Phylogenetic tree of selected OMT peptides. OMTs in bold are known to act in the methylation of anthocyanins. OMT names and GenBank accession numbers are as follows: *Fuchsia*, FMT, HB975539; *Vitis vinifera*, VvAOMT, BQ796057; *Torenia*, TMT5, HB975529; *Petunia* difE, FMT, HB975519; *Cyclamen persicum×Cyclamen purpurascens*, CkmOMT2, BAK74804; *Mesembryanthemum crystallinum*, McPFOMT, AY145521; *Stellaria longipes*, SlCCoAOMT, L22203; *Glycine max*, GmAOMT, ADX43927; *Arabidopsis thaliana*, AtCCoAOMT, AAM64800; *Zea mays*, ZmCCoAOMT, AJ242980; *Medicago sativa*, MsCCoAOMT, AAC28973; *Vitis vinifera*, VvCCoAOMT, Z54233; *Nicotiana tabacum*, NtCCoAOMT, U38612; *Populus balsamifera* subsp., PtCCoAOMT, AJ224896; *Homo sapiens*, HOMT, A38459; *Medicago sativa*, MsIOMT, AAC49927; *Catharanthus roseus*, CrAOMT, AY127568; *Oryza sativa*, OsROMT, DQ288259; *Thalictrum tuberosum*, TtOMT, AF064693; *Nicotiana tabacum*, NtCOMT, AF484252; *Vitis vinifera*, VvCOMT, AF239740; *Medicago sativa*, MsCOMT, M63853; *Chrysosplenium americanum*, CaCOMT, AAA86982 and CaAOMT, U16794. The number beside the branches represents the bootstrap values based on 1000 replicates using MEGA 5. Bar, nucleotide substitutions per site.

To confirm our hypothesis, two genes with ORFs of 708bp that encoded for methyltransferase were obtained from *P. suffruticosa* cv. ‘Gunpohden’ and *P. tenuifolia*. The deduced amino acid sequences of these genes contained a conserved domain similar to those of the SAM or AdoMet_MTases superfamily (cl17173) and the Methyltransf_3: *O*-methyltransferase superfamily (pfam01596), and were designated *PsAOMT* and *PtAOMT*, respectively. The putative peptides of these proteins had 235 aa and a calculated molecular mass of 26.4kDa, and they each contained a conserved structural fold of seven β-sheets (Fig. 3) (Cheng and Roberts, 2001). Surprisingly, there were a total of four amino acids (positions 13, 85, 87, and 205) that differed between PsAOMT and PtAOMT (Fig. 3). A BLAST analysis indicated that PsAOMT and PtAOMT were homologous to several previously characterized type II subclass OMTs. PsAOMT shared 73% identity with VvAOMT from grapevine (Hugueney *et al.*, 2009), 62% with GmAOMT from *Glycine max* (Kovinich *et al.*, 2011), and 58% with PFOMT from *M. crystallinum* (Ibdah *et al.*, 2003). Phylogenetic analysis demonstrated that PsAOMT belongs to the type IIOMTs (with a low molecular weight and Mg^2+^ dependence) and was similar to the typical example of this class of enzyme, CCoAOMT (Fig. 4). A subclass of the type II OMTs specific for flavonoid OMTs has been proposed (Ibdah *et al.*, 2003), and thus PsAOMT is a possibly new member of this subclass together with VvAOMT, McPFOMT, and several other polypeptides described in a patent (Brugliera *et al.*, 2003) (Fig. 3).

### Biochemical characterization of recombinant AOMTs

The observed molecular weight of both recombinant PsAOMT and PtAOMT was approximately 66kDa (comprising 26kDa of target protein plus 40kDa of maltose-binding protein tag) (Supplementary Fig. S4, available at *JXB* online). The reaction conditions for purifying recombinant PsAOMT was optimized, and the results demonstrated that PsAOMT activity was completely absent when EDTA was present (Fig. S5, available at *JXB* online), indicating that it was cation dependent. We also tested the influence of five divalent cations (Mg^2+^, Ca^2+^, Mn^2+^, Co^2+^, and Zn^2+^) on PsAOMT activity with the substrate Qu3R. In comparison with the other four divalent cations, PsAOMT showed the highest activity in the presence of Mg^2+^, and the optimal Mg^2+^ concentration was 1.0mM (Supplementary Fig. S5). Recombinant PsAOMT was active within a pH range of 6.5–8.5, with an optimum of approximately 7.5 with the substrate Qu3R (Supplementary Fig. S5).

The activities of purified recombinant PsAOMT and PtAOMT were analysed by using a number of potential substrates including anthocyanidins, anthocyanins, flavonols, flavones, flavan-3-ols, and phenolic acid based on optimized conditions (Table 1, Supplementary Figs S1 and Fig. S6, available at *JXB* online). PsAOMT could use anthocyanins as methoxyl accepters, and they acted to methylate the 3′-hydroxyl group of the B-ring with high affinity and efficiency. Pg3G was the only tested anthocyanin compound that was not a substrate for PsAOMT. With Dp3G, a sequential methylation occurred at the 3′- and 5′-hydroxyl group on the B-ring (Table 1 and Supplementary Table S3, available at *JXB* online). In comparison with the substrates Dp3G, Qu3R, and quercetin, PsAOMT had a higher affinity for Cy3G and Cy3G5G (Table 1), suggesting that cyanidin-derived anthocyanins are high-affinity substrates for PsAOMT. Unlike anthocyanins, anthocyanidin cyanidin could be used as substrate by PsAOMT with a low activity. In addition, we could not detect the methylated products of the anthocyanidin delphinidin (Supplementary Table S3), which suggested that delphinidin might be unstable in the reaction system, or might be due to the low enzymatic activity of PsAOMT. PsAOMT could also methylate Lu7G and Lu with weak affinity. The following substrates could not be methylated by PsAOMT: Km3G, kaempferol, luteolin, apigenin, naringenin, epicatechin, and caffeic acid (Table 1). Surprisingly, although PtAOMT possesses identical substrate specificity and biochemical properties, the catalytic activity of PtAOMT was much lower than that of PsAOMT (approximately 60-fold lower) (Table 1).

**Table 1.** Overview of PsAOMT and PtAOMT activities with various substrates under optimized assay conditions

Substrate	PsAOMT					PtAOMT
	*K*_m_ (μM)	*V*_max_ (nM^–1^)	*K*_cat_× 10^–3^ (s^–1^)	*K*_cat_ */K*_m_ (M^–1^s^–1^)	Specific activity (pkat mg^–1^)	Specific activity (pkat mg^–1^)
Pelargonidin 3-*O*-glucoside	–	–	–	–	–	–
Cyanidin 3,5-di-*O*-glucoside	1.06 (0.01)	17.88 (0.25)	127.70 (1.05)	120886 (563)	1788 (15)	29.60 (3.47)
Cyanidin 3-*O*-glucoside	1.76 (0.02)	11.57 (0.05)	161.99 (0.73)	91829 (421)	2314 (11)	37.98 (1.05)
Delphinidin 3-*O*-glucoside	4.11 (0.35)	17.05 (0.27)	94.72 (1.49)	23293 (1538)	1364 (21)	21.50 (0.10)
Quercetin 3-*O*-rutinoside	12.32 (0.32)	27.44 (1.63)	192.11 (4.41)	15599 (110)	2744 (135)	30.20 (0.24)
Luteolin 7-*O*-glucoside	146.26 (10.07)	31.61 (1.64)	0.22 (0.00)	1486 (145)	2017 (109)	2.24 (0.18)
Kaempferol 3-*O*-glucoside	–	–	–	–	–	–
Cyanidin	–	–	–	–	0.40 (0.05)	–
Delphinidin	–	–	–	–	–	–
Quercetin	4.33 (0.07)	1.77 (0.02)	9.83 (0.12)	2271 (24)	142 (2)	17.58 (0.57)
Luteolin	–	–	–	–	0.043 (0.002)	2.08 (0.27)
Kaempferol	–	–	–	–	–	–
Apigenin	–	–	–	–	–	–
Naringenin	–	–	–	–	–	–
Epicatechin	–	–	–	–	–	–
Caffeic acid	–	–	–	–	–	–

### A single amino acid substitution can dramatically alter the catalytic activity of AOMTs

The high amino acid sequence identities of PsAOMT and PtAOMT indicated that their divergent enzymatic activity might result from differences in amino acid residues at four positions. Four separate site-directed mutations were constructed by using the low-activity enzyme PtAOMT as a template, namely PtAOMT-G13E, PtAOMT-T85A, PtAOMT-R87L, and PtAOMT-T205R. When assayed for 5min under the optimized reaction conditions with Cy3G5G as a substrate, only Pn3G5G was detected from the PtAOMT-R87L reaction product, and the observed *in vitro* catalytic efficiency of PtAOMT-R87L was equal to that of PsAOMT (Table 2). To further characterize these mutations, the reaction durations were extended from 5 to 30min, and the recombinant G13E, T85A, and T205R enzymes displayed 1.4, 4.1, and 9.1%, respectively, of the PsAOMT catalytic activity (100%). The PtAOMT activity was 1.5% of that of PsAOMT (100%), and the increased duration of the assays did not lead to a detectable increase in PtAOMT activity (Table 2). However, the single R87L mutation of PtAOMT led to an equally high activity (104.5%) as that of PsAOMT (100%). This result indicated that a leucine residue at position 87 plays an important role in the catalytic activity of these AOMTs. To validate that a leucine at position 87 was indeed a key amino acid residue, two mutations (L87R and L87A) of PsAOMT were constructed. Both these mutant enzymes exhibited decreased catalytic activity (similar to the activity of recombinant PtAOMT) (Table 2). A kinetic study of PtAOMT-R87L showed that its substrate specificity was identical to that of PsAOMT and its specific activity was similar to that of PsAOMT (Supplementary Table S4, available at *JXB* online). It is worth noting that the *K*_m_ of PtAOMT-R87L for Cy3G was slightly lower than its *K*_m_ for Cy3G5G; the opposite was true for PsAOMT. These results demonstrated that Leu-87 is a critically important residue for the catalytic activity of these AOMT enzymes and that a single amino acid mutation can cause a dramatic decrease in activity without a loss of function.

**Table 2.** Comparison of catalytic activities of the recombinant AOMTs generated using site-directed mutagenesis, using Cy3G5G as the substrate Activity (%): recombinant protein activity as a percentage of the ‘full’ activity measured for PsAOMT; SD, standard deviation with three repetitions.

Polypeptide	Incubation for 5min [% (±SD)]	Incubation for 30min [% (±SD)]
PsAOMT	100(0)	100(0)
PtAOMT-G13E	0(0)	1.4(0.1)
PtAOMT-T85A	0(0)	4.1(0.3)
PtAOMT-R87L	100.7(1.1)	104.5(0.7)
PtAOMT-T205R	0(0)	9.4(0.5)
PsAOMT-L87R	0(0)	4.0(0.2)
PsAOMT-L87A	0(0)	4.8(0.4)
PtAOMT	0(0)	1.5(0.3)

### Structural basis for substrate discrimination in AOMTs

**Fig. 5.**
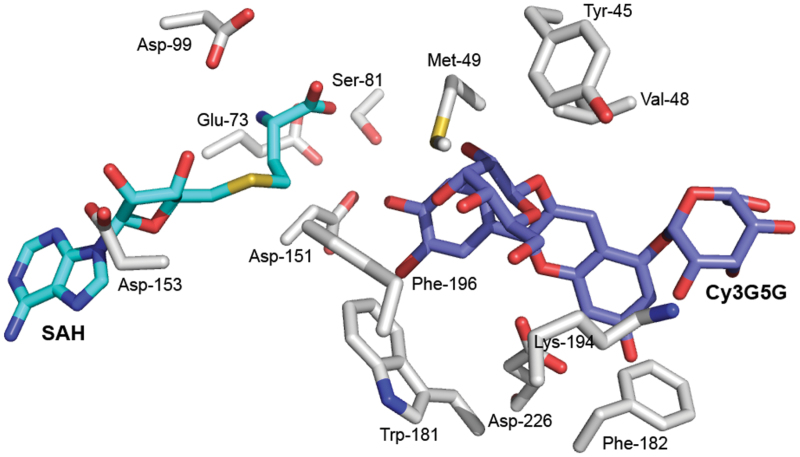
Molecular model of the PsAOMT active site. The SAH (*S*-adenosyl-l-homocysteine), Cy3G5G, and substrate-binding residues are represented as sticks, and labelled in cyan, grey, and white, respectively. The oxygen, nitrogen, and sulfur atoms are labelled in red, blue, and yellow, respectively.

The 3D models of PsAOMT and PtAOMT were computed and analysed to investigate the structural basis for their activity differences (Supplementary Fig. S7, available at *JXB* online). The SAM-binding residues (Met-49, Glu-73, Ser-81, Asp-99, Asp-151, and Asp-153) were conserved in both OMTs and were very similar to those of CCoAOMT (Fig. 5). After careful analyses of the PsAOMT model and the substrate-binding site of CCoAOMT, the residues Tyr-45, Val-48, Trp-181, Phe-182, Lys-194, Phe-196, and Asp-226 were predicted to be the putative substrate-binding pocket of Cy3G5G (Fig. 5). To our surprise, the single amino acid residue (Leu-87) that contributes the substrate specificity of PsAOMT is far from the putative substrate-binding pocket (Supplementary Fig. S7).

### *In vivo* characterization of AOMTs

**Fig. 6.**
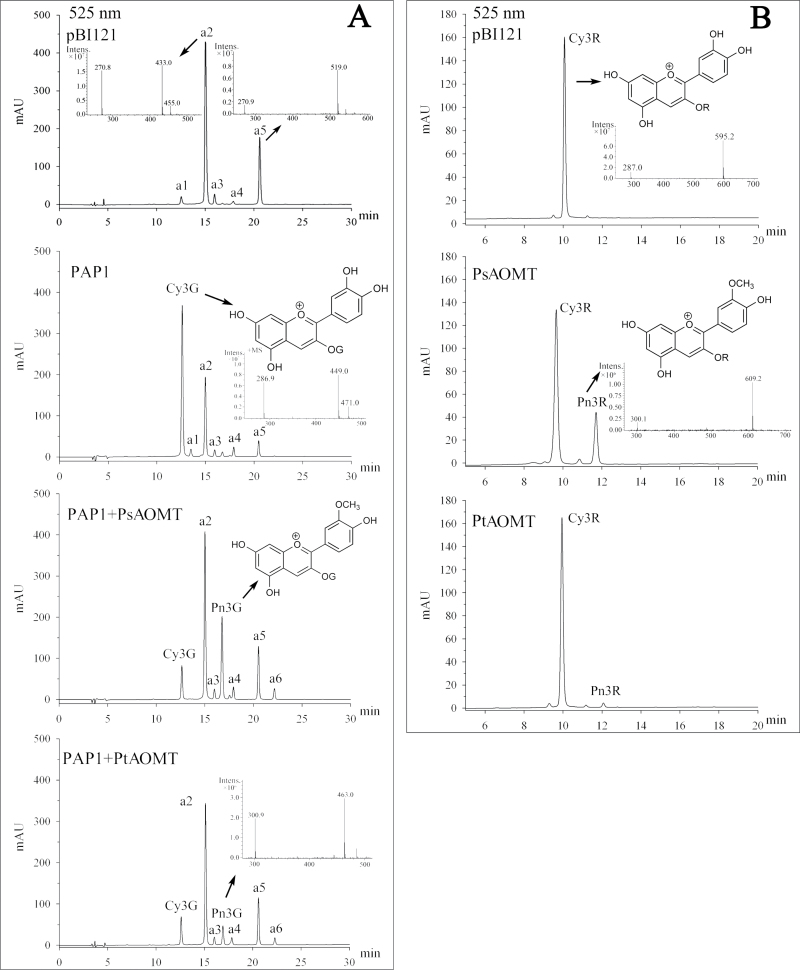
Characterization of AOMT activity *in vivo* using transient expression in strawberry and the stable transformation of tobacco. (A) Anthocyanin profiles of strawberry fruits with transient *PBI121* or *PAP1* expression, and the co-expression of *PAP1/PsAOMT*, and *PAP1/PtAOMT* (4 d of expression). Other anthocyanins are shown in Supplementary Table S5, available at *JXB* online. (B) Anthocyanins from transgenic tobacco flowers (35S::*PsAOMT*, 35S::*PtAOMT* and empty vector control, respectively) were analysed by HPLC-MS.

We first investigated the catalytic activities of the *Paeonia* AOMTs *in vivo* by using transient expression in strawberry fruits. The strawberry fruit mostly contained the pelargonidin type of anthocyanins (da Silva *et al.*, 2007), which are known for not being AOMT substrates. To provide appropriate substrates for the enzymatic reactions, the anthocyanin biosynthesis-related R2R3 MYB transcription factor *PAP1* from *Arabidopsis* (Borevitz *et al.*, 2000) was introduced along with the two *Paeonia AOMT* sequences by agroinfiltration (Hoffmann *et al.*, 2006). The simultaneous expression of *PAP1* and *AOMT* transcripts resulted in the synthesis of Cy3G (*m*/*z* 449 [M]^+^) and Pn3G (*m*/*z* 463 [M]^+^) in strawberry fruits (Fig. 6A). It should be noted that there are several anthocyanin compounds that occur naturally in strawberries (a1– a6 shown in Supplementary Table S5). Pn3G accumulated to higher levels than those of Cy3G in the *PAP1* and *PsAOMT* transformants. Conversely, the Cy3G levels were higher than the Pn3G levels in the *PAP1* and *PtAOMT* transformants (Fig. 6A).

Next, we used *Agrobacterium*-mediated transformation of tobacco for *in vivo* studies of AOMT function. Tobacco has pink flowers that are known to contain cyanidin-3-*O*-rutinoside (Cy3R). Five and four transgenic tobacco lines were found to accumulate similar mRNA levels of *PsAOMT* and *PtAOMT* (Supplementary Fig. S8, available at *JXB* online), respectively. The anthocyanin profiles of these transgenic and control lines were analysed by HPLC-MS (Fig. 6B). The results demonstrated that five 35S::*PsAOMT* lines accumulated peonidin-3-*O*-rutinoside (Pn3R), with a peak of *m*/*z* 609 [M]^+^, accounting for 21.7±1.8% of total anthocyanin, and Pn3R was also detected in four 35S::*PtAOMT* transgenic lines, accounting for a lower 1.9±0.2% total anthocyanin content. This finding indicated that the proteins encoding *PsAOMT* and *PtAOMT* had a contrasting catalytic activity *in vivo*, which was consistent with the characterization of recombinant proteins *in vitro*.

**Fig. 7.**
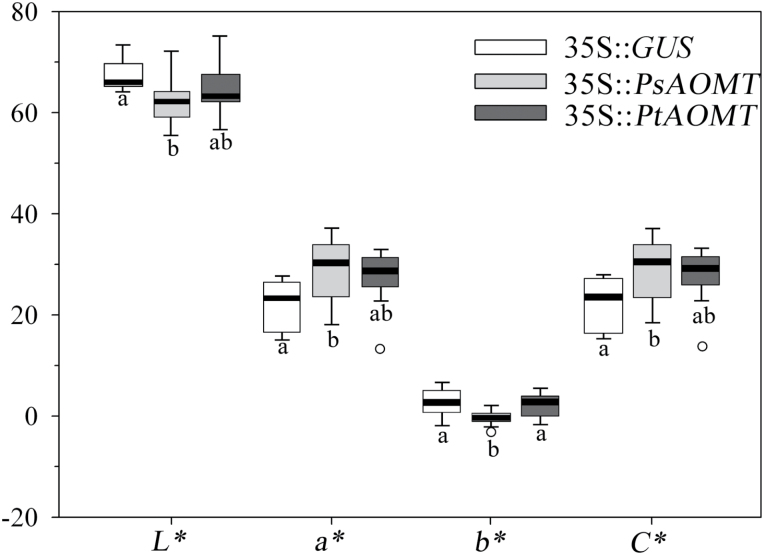
CIELab colour space analysis of transgenic tobacco flower colour. L* represents lightness, from black (0) to white (100); a* represents red (positive) to green (negative); b* represents yellow (positive) to blue (negative); and C* represents the chroma or saturation of the colour. Boxes of each parameter with no common letter in the figure body indicate a significant difference at *P*<0.05 (*n*≥9).

To evaluate the influence of the introduced *PsAOMT* and *PtAOMT* on flower coloration, chromatic analyses of transgenic tobacco lines and controls were performed. One-way analysis of variance was performed for the colour parameters L*, a*, b*, and C*, and significant differences were observed between 35S::*PsAOMT* and control flowers (Fig. 7). The 35S::*PsAOMT* transgenic lines had higher values of a* towards red, and lower values of *b* ^*^ towards blue, leading to an *h* change towards a purple hue compared with that of the control, which demonstrated that methylated anthocyanins would resulted in purplish flower colours. The colour parameters of the 35S::*PtAOMT* lines fell in between those of the 35S::*PsAOMT* lines and the control and showed no significant differences among them (Fig. 7).

This result further validated the OMT activity of AOMTs of *Paeonia* and underscored the effects that dramatically different enzymatic efficiencies can cause, leading to differences in the biosynthesis and accumulation of these compounds.

### *AOMT* expression patterns are consistent with anthocyanin accumulation

To determine whether there was any correlation between the expression levels of *AOMT* transcripts and flavonoid accumulation during floral development in *Paeonia* spp., the corresponding flower petals from S1–S5 were harvested and assayed for the expression of *AOMT* transcripts. The results indicated that the *PsAOMT* expression levels in *P. suffruticosa* cv. ‘Gunpohden’ were consistent with the anthocyanin accumulation levels in petals. As shown in Fig. 2, anthocyanins were barely detected at the beginning of the colourless stage (S1), and *PsAOMT* transcripts were also detected at low levels (Fig. 2). From the flower coloration stage onward, the *PsAOMT* expression level increased and reached a maximum when the flower bud became loose and ready to bloom (S3) (Fig. 2). The methylated anthocyanin Pn3G5G accumulated to its maximum amount at S5 because of the continuous expression of *PsAOMT*. The expression levels of *PsAOMT* showed no obvious relationship with the accumulation of flavones and flavonols because the methylated colourless flavonoids (Is/Ch glycosides) already existed before the onset of S1 (Supplementary Fig. S3) when *PsAOMT* transcripts were hardly detected. The concentrations of these compounds did not change with the increasing expression levels of *PsAOMT* transcripts (Fig. 2 and Supplementary Fig. S3). These results indicated that *PsAOMT* was related to and specific for methylated anthocyanin accumulation in *Paeonia*. The expression pattern of *PtAOMT* in *P. tenuifolia* was identical to that of *PsAOMT* in *P. suffruticosa* cv. ‘Gunpohden’ (Fig. 2). However, there was no noticeable correlation between the *PtAOMT* expression levels and the accumulation of methylated anthocyanin compounds (Fig. 2 and Supplementary Fig. S3). We inferred that this finding was caused by the relatively weak enzyme activity of PtAOMT in comparison with that of PsAOMT.

In addition, *AOMT* expression was evaluated in various vegetative organs (Fig. 2). *PsAOMT* transcripts could be detected at very low levels in roots and stems, and they were detected at slightly higher levels in leaf and sepal tissues, which are known to accumulate low anthocyanin concentrations. *PtAOMT* transcripts were detected in the roots and leaves at very low levels and were noticeable in the stems and sepals.

### AOMT catalyses the biosynthesis of methylated anthocyanins and leads to purple coloration in *Paeonia* flowers

We sequenced *AOMT* cDNA from five tree peony cultivars (‘Fengdan’, ‘Hongling’, ‘Liuguangyicai’, ‘Chaoyi’, and ‘Qinglongwomochi’), two herbaceous peony cultivar (‘Dafugui’ and ‘Zixiayingri’), and one intersectional hybrid (‘Hexie’) between tree peony and herbaceous peony, which all have a red/purple or purple flower colour (Supplementary Table S2, Supplementary Fig. S9, available at *JXB* online). The *AOMT* genes obtained from these plants were nearly identical, and all of them possessed the critically important leucine residue at position 87 (Supplementary Table S2, Supplementary Fig. S10, available at *JXB* online). A chemical analysis of anthocyanins from the five cultivars by HPLC techniques showed that methylated anthocyanins (peonidin glycosides) accounted for 87–95% of the total anthocyanins (Supplementary Table S2, Supplementary Fig. S11, available at *JXB* online) and cyanidin glycosides were detected as minor compounds, indicating that all the cultivars possessed efficient methyltransferases to catalyse the methylation of cyanidin glycosides to peonidin glycosides. As a result, peonidin glycosides accumulated in the petals, leading to the purple coloration of *Paeonia* flowers. These findings reinforced the notion that AOMTs with leucine residues at position 87 contribute to purple flower coloration in *Paeonia* plants.

## Discussion

### Functional characterization of AOMTs from *Paeonia* and their active switch

A phytochemical analysis in *Paeonia* plant petals on methylated anthocyanin compounds accumulation suggested that strong methylation activity occurs in the petals (Wang *et al.*, 2001; Zhang *et al.*, 2007; Jia *et al.*, 2008; Li *et al.*, 2009). Therefore, we identified and characterized genes in this study for anthocyanin methyltransferases that belonged to the new type II OMTs. The characterization of recombinant PsAOMT and PtAOMT with a wide range of flavonoid compounds and caffeic acids as substrates demonstrated that they both methylated flavonoids with a vicinal dihydroxy on the B-ring and a 3-hydroxyl on the C-ring. The kinetic parameters of PsAOMT showed that the specific activities of anthocyanins and flavonols were similar to their activity in VvAOMT, and the *K*_m_ values for anthocyanins were smaller than those in VvAOMT (Hugueney *et al.*, 2009), displaying a better affinity with the optimum substrates. In addition, PsAOMT showed much lower catalytic activities with anthocyanidins, quercetin, and luteolin, indicating that it was able to methylate glycosylated flavonoids rather than aglycones. In peony flowers, the major anthocyanins are both methylated and glycosylated, and the order of aglycone modifications *in vivo* remain unknown. The present work suggested that methylation may occur after glycosylation during anthocyanin biosynthesis, which is consistent with reports in grape berries (Ford *et al.*, 1998; Hugueney *et al.*, 2009). To clarify the order of methylation and glycosylation *in vivo*, the further characterization of the flavonoid glycosyltransferase in *Paeonia* will be meaningful.

The dimer of type II OMTs lacking a domain for dimerization in the N-terminal region could be formed by hydrophobic interactions. Each monomer could interact with substrate(s) and cofactor(s), unlike type I OMTs, which require a homodimeric structure to perform methylation (Noel *et al.*, 2003). Surprisingly, we found that the leucine at position 87 of PsAOMT was vital for the high activity level, and a mutant with an arginine residue at the same position in PsAOMT led to a remarkable decrease in enzymatic activity.

OMT-catalysed methylation reactions require a methyl donor (SAM), a methyl accepter (substrate), and an appropriately conformed reaction centre. Typically, the SAM-binding residues comprise a highly glycine-rich sequence of E/DXGXGXG known as motif I, which is highly conserved in the N-terminal region of OMTs (Martin and McMillan, 2002). The N terminus and a variable insertion loop near the C terminus played important roles in the substrate specificity (Kopycki *et al.*, 2008). The key amino acid at position 87 was located in the α4 helix proximal to motif I (Fig. 3), suggesting that a mutation in this amino acid may influence the binding of SAM or the methyl transfer. Although the PtAOMT and PsAOMT activities differ greatly in terms of catalytic efficiency, these enzymes shared the same substrate specificities, indicating that the substrate-binding regions of both proteins were probably conserved. The SAM-binding site might be altered by the single amino acid substitution at position 87 in the two proteins, and this mutation may be responsible for the observed differences in the enzyme turnover rates but may not affect substrate regioselectivity. The substitution may also affect the methylation reaction. The currently recognized catalytic mechanisms of OMTs are S_N_2-like reactions, which are metal dependent, acid/base residues, or mediation by proximity and desolvation (Liscombe *et al.*, 2012). The arginine residue is polar, unlike leucine (a neutral amino acid), which may change the hydrophobic environment around SAM or the catalytic centre. However, Leu-87 of PsAOMT is far from the putative substrate-binding pocket; however, mutations at distant residues can alter the properties of the active site region and change the activity as reported for glutathione *S*-transferase (Ketterman *et al.*, 2001), alcohol dehydrogenase (Nagel *et al.*, 2013), and regulator proteins (Freeman *et al.*, 2011). Mutations at distant residues might cause dynamic motion at the active site that is involved in the conformational change of the substrate-binding region and then alter the catalytic efficiency and substrate specificity. To elucidate the mechanism precisely, X-crystal structures of AOMTs must be further investigated.

### AOMTs are primarily responsible for anthocyanin methylation in peonies

There were four major anthocyanins and nine major colourless flavonoids in peony flowers in this study. The anthocyanins consisted of two anthocyanidins, namely, non-methylated cyanidin and monomethylated peonidin, with mono- or diglucosylation. The colourless flavonoids included glycosides derived from non-methylated quercetin, luteolin, apigenin, kaempferol, monomethylated isorhamnetin and chrysoeriol (Li, *et al.*, 2009). Anthocyanins (Fig. 2 and Supplementary Fig. S11) and the other six colourless flavonoids (Supplementary Fig. S3) as well as *AOMT* expression were investigated during flower development. The results indicated that *PsAOMT* expression was notably consistent with the accumulation of monomethylated peonidin glycosides in *P. suffruticosa* cv. ‘Gunpohden’ flowers. In contrast, peonidin glycosides were barely accumulated in *P. tenuifolia* flowers, because of weak PtAOMT activity, while the AOMT activity and accumulation of methylated colourless flavonoids seemed less relevant. Although recombinant PsAOMT could employ flavonols and flavones as substrates *in vitro* (Table 1), the methylated colourless flavonoids in tree peony flowers were minor components, such as Is3G7G, Ch7G, and Ch7Neo (Supplementary Fig. S3). Moreover, these methylated colourless flavonoids were detected at early stages before colouration, and the content increased slowly, displaying no obvious correlation with the *PsAOMT* expression. This finding might be explained by the specificity of the substrate structure and anthocyanin competition, or there may be other flavone and flavonol OMTs in peonies.

### Methylation of anthocyanins is involved in the purplish flower colouration of *Paeonia* spp.

In ornamental plants, the mechanism of blue flower colouration has been extensively studied, and consists primarily of delphinidin anthocyanins (Yoshida *et al.*, 2009). However, the basis of coloration of purple flowers from cyanidin-based anthocyanins remains unclear at present. Cyanidin-based anthocyanins confer red/purple and purple colours to plants, with some exceptions such as cornflower (a blue colour) (Shiono *et al.*, 2005). The flower colour phenotype is controlled primarily by pigment chemical structures and the concentrations of particular pigments (Nakayama *et al.*, 2012). In the present study, the two plant materials presented purple and vivid red colours, although they contained the same four cyanidin-based derivatives and similar total concentrations of anthocyanins. The vast difference in methylated anthocyanins (peonidin glycosides) was dominant in purple flowers and for cyanins as the major pigment in red flowers. This finding suggested that methylation varied the colour of cyanidin-based anthocyanins from red to purple. To elucidate the influence of methylation on coloration, transgenic tobacco lines with overexpressed *PsAOMT* were generated. The flower colour of transgenic tobacco plants with different methylation profiles corresponding to those in Fig. 6 demonstrated significant changes in chromatic parameters towards a purple hue, compared with that of the control (Fig. 7). A similar phenomenon was reported in fragrant cyclamen (Akita *et al.*, 2011). A purple-flowered fragrant cyclamen lacking an enzyme for anthocyanin OMT (*CkmOMT2*) (ion-beam irradiation deletion) produced red/purple flower phenotypes (Akita *et al.*, 2011).

Sakata *et al.* (1995) studied 38 tree peony cultivars in Japan and concluded that hydroxylation and methylation both contributed considerably to the blueing of flower colours. Chemical and chromatic analyses of hundreds of tree peony cultivars showed that the methylation level of anthocyanins was significantly correlated with the purpleness of the flower colour (Li, 2010). Supplemental information including the flower colour, chromatic parameters, anthocyanin methylation levels and the key amino acid of corresponding AOMTs for 10 typical purple or red-flowered cultivars are summarized in Supplementary Table S2. Taken together, these findings show that there was a noticeable correlation for cultivars that contained a key leucine amino acid and then generated a high level of methylated anthocyanins, resulting in a purple flower.

In comparison with the methylation of anthocyanins in tree peony, the minor differences in glycosylation might play less of a role in flower coloration because it may simply increase the solubility or decrease anthocyanic vacuolar inclusions (Morita *et al.*, 2005). The former study demonstrated that, in tree peony flowers, glycosylation was present only with mono- and diglucosides, and the number of glucose moieties did not contribute to the blueing of the tree peony flower (Sakata *et al.*, 1995; Li, 2010).

Co-pigments (colourless flavonoids) are another difference between the two plant materials. Other flavonoid compound profiles of *P. suffruticosa* cv. ‘Gunpohden’ and *P. tenuifolia* flowers were analysed at 350 and 280nm (Supplementary Fig. S2). Red flowers contained negligible amounts of co-pigments, and co-pigments were abundant in purple flowers (Supplementary Fig. S2). Co-pigments interacting with anthocyanins at the proper concentrations and ratios can result in a bathochromic shift (Asen *et al.*, 1971). However, a study of Japanese tree peony cultivars showed that there were pink-flowered cultivars with high co-pigment contents and purple-flowered cultivars with low co-pigment contents, suggesting that co-pigmentation might not be a major factor in the blueing of tree peony flowers (Sakata *et al.*, 1995). As the content of flavonols increased, the blue flower of lisianthus changed towards red (Nielsen *et al.*, 2002). Flavone accumulation in transgenic torenia made the flower bluer (Aida *et al.*, 2000). Nevertheless, these studies were based primarily on blue flowers with delphinidin-based anthocyanins. Whether these compounds influence coloration in flowers with cyanidin-based anthocyanins, and the mechanism of any such influence, will require further investigation.

Similar chemical and chromatic analyses of lotus cultivars have also been investigated, and the results suggested that the methylation level of anthocyanins was significantly correlated with the hue towards purpleness (Yang, 2009; Yang *et al.*, 2009). Heredia *et al.* (1998) reported a chromatic characterization of five anthocyanins from red grape skins obtained in a spectroscopic study, and showed that as the degree of methylation increased, a shift towards purple was observed. This finding suggested that anthocyanin methylation might commonly contribute to purple coloration, especially in cyanidin-based pigment systems.

In conclusion, we characterized an AOMT in *Paeonia* plants. Based on *in vitro* and *in vivo* experiments, we grouped this AOMT into a recently defined subclass of type II OMTs and identified the specificity for anthocyanin substrates. In addition, we characterized the biochemical properties of recombinant PsAOMT and found that a mutation in a key amino acid residue could dramatically change the catalytic efficiency. Moreover, the AOMT is likely to be responsible for the methylation of anthocyanins and to contribute to the purple coloration of flowers in *Paeonia* plants. Thus, research on AOMTs in *Paeonia* is an important step for understanding anthocyanin modifications, and their contribution to flower coloration innovation is relevant for ornamental plants.

## Supplementary data

Supplementary data are available at *JXB* online.

Supplementary Fig. S1. The chmical structure of substrates used for *in vitro* analysis of recombinant PsAOMT and PtAOMT.

Supplementary Fig. S2. HPLC profiles of flower extracts from *Paeonia suffruticosa* cv. ‘Gunpohden’ (A) and *Paeonia tenuifolia* (B) at 350 and 280nm.

Supplementary Fig. S3. The accumulation of colourless flavonoids in *Paeonia suffruticosa* cv. ‘Gunpohden’ petals during flower development.

Supplementary Fig. S4. SDS-PAGE of recombinant PsAOMT.

Supplementary Fig. S5. The catalytic activity of PsAOMT with quercetin 3-*O*-rutinoside (Qu3R) as a substrate under different reaction conditions, including a pH gradient of buffer solutions, the presence of different divalent cations, and various Mg^2+^ concentrations.

Supplementary Fig. S6. Profiles of products methylated by recombinant PsAOMT using a series of substrates in the *in vitro* system by HPLC analysis.

Supplementary Fig. S7. Superimposed 3D models of PsAOMT and PtAOMT represented as cartoons.

Supplementary Fig. S8. The expression of *AOMT*s in transgenic tobacco lines according to RT-PCR.

Supplementary Fig. S9. Flower phenotypes of different *Paeonia* cultivars used in this study.

Supplementary Fig. S10. Amino acid sequence alignments of the AOMTs from 10 *Paeonia* plants.

Supplementary Fig. S11. HPLC profiles of anthocyanins in flower petals.

Supplementary Table S1. The primer sequences used in site-directed mutagenesis and qPCR.

Supplementary Table S2. A summary of the 10 specimens, including information regarding the flower colour, chromatic parameters, total anthocyanin content, methylation levels of the anthocyanins, and the key amino acid(s) of the AOMTs.

Supplementary Table S3. Identification of products methylated by recombinant PsAOMT using a series of substrates in the *in vitro* system.

Supplementary Table S4. PtAOMT-R87L activities with major substrates under optimized assay conditions.

Supplementary Table S5. Anthocyanins in strawberry fruits as detected by HPLC-MS (demonstrated in Fig. 6).

Supplementary Data

## Abbreviations:

3D: three-dimensional
AOMT: anthocyanin *O*-methyltransferase
Ch7Neo: chrysoeriol 7-*O*-neohesperidose
Cy3G: cyanidin 3-*O*-glucoside
Cy3G5G: cyanidin 3,5-di-*O*-glucoside
DAD: diode-array detection
Dp3G: delphinidin 3-*O*-glucoside
DW: dry weight
ESI: electrospray ionization
HPLC: high-performance liquid chromatography
Is3G7G: isorhamnetin 3,7-di-*O*-glucoside
Km3G: kaempferol 3-*O*-glucoside
Lu7G: luteolin 7-*O*-glucoside
Lu7Neo: luteolin 7-*O*-neohesperidose
MS: mass spectrometry
OMT: *O*-methyltransferase
ORF: open reading frame
Pg3G: pelargonidin 3-*O*-glucoside
Pn3G: peonidin 3-*O*-glucoside
Pn3G5G: peonidin 3,5-di-*O*-glucoside
qPCR: quantitative PCR
Qu3G7G: quercetin 3,7-di-*O*-glucoside
Qu3R: quercetin 3-*O*-rutinoside
SAM: *S*-adenosylmethionine.
