# Supplementary material for: Methylation mediated by an anthocyanin, O-methyltransferase, is involved in purple flower coloration in Paeonia
Source: J Exp Bot. 2015 Jul 23;66(21):6563–77. doi: 10.1093/jxb/erv365 (PMC4623676; doi:10.1093/jxb/erv365)
Supplement: Supplementary Data [file supp_66_21_6563__index.html]

Methylation mediated by an anthocyanin, O-methyltransferase, is involved in purple flower coloration in Paeonia — Supplementary Data 

# Methylation mediated by an anthocyanin, *O*-methyltransferase, is involved in purple flower coloration in *Paeonia*

## Supplementary Data

Data files

- Supplementary Data - Supplementary Data
- Supplementary Data - Supplementary Data
