# Supplementary material for: Methylation mediated by an anthocyanin, O-methyltransferase, is involved in purple flower coloration in Paeonia
Source: J Exp Bot. 2015 Jul 23;66(21):6563–77. doi: 10.1093/jxb/erv365 (PMC4623676; doi:10.1093/jxb/erv365)
Supplement: Supplementary Data [file supp_erv365_jexbot149625_file002.pdf]

# Methylation mediated by an anthocyanin *O*-methyltransferase is involved in purple flower coloration in *Paeonia*

Hui Du, Jie Wu, Kui-Xian Ji, Qing-Yin Zeng, Mohammad-Wadud Bhuiya, Shang Su,

Qing-Yan Shu, Hong-Xu Ren, Zheng-An Liu and Liang-Sheng Wang

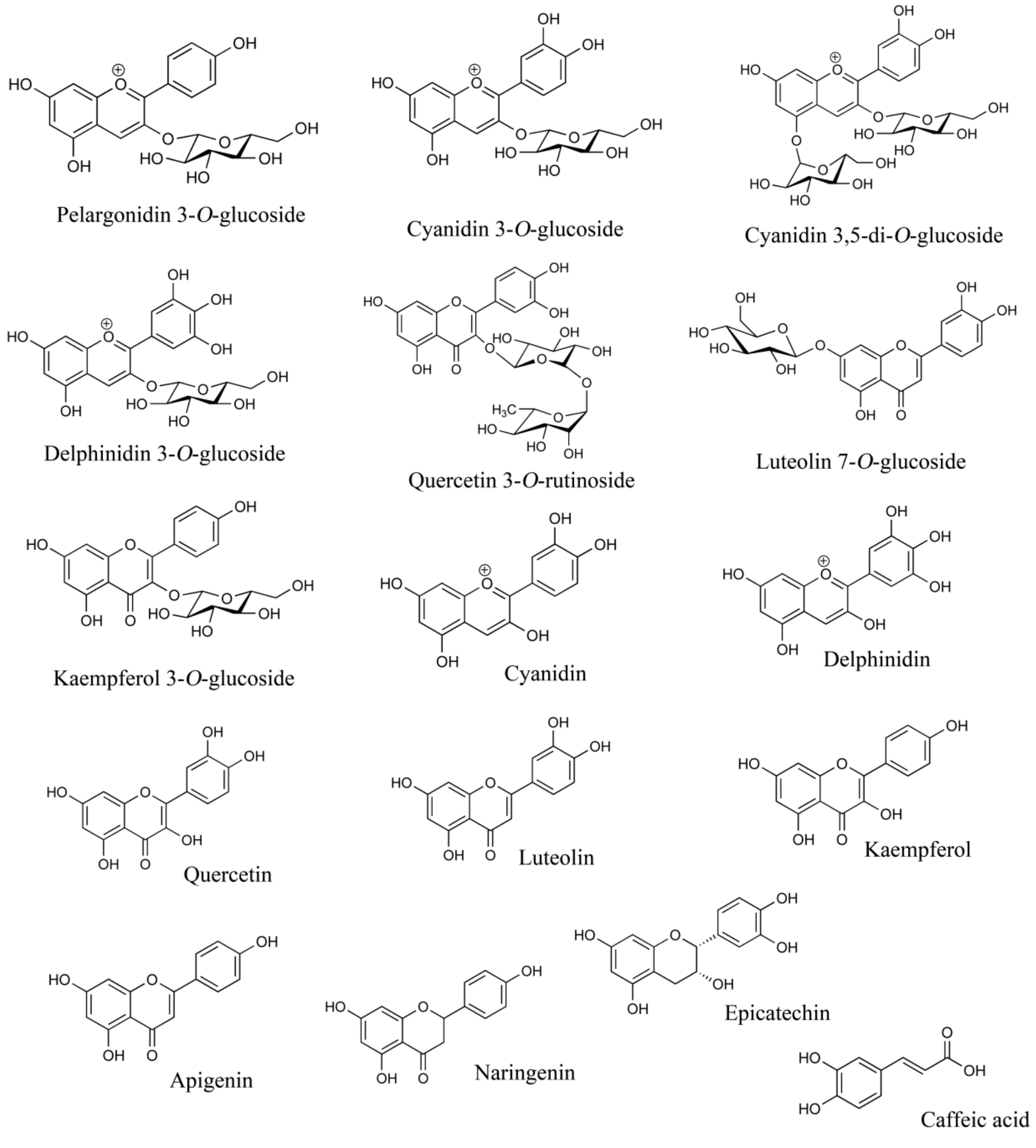


**Figure S1.** The chmical structure of substrates used for *in vitro* analysis of recombinant PsAOMT and PtAOMT.

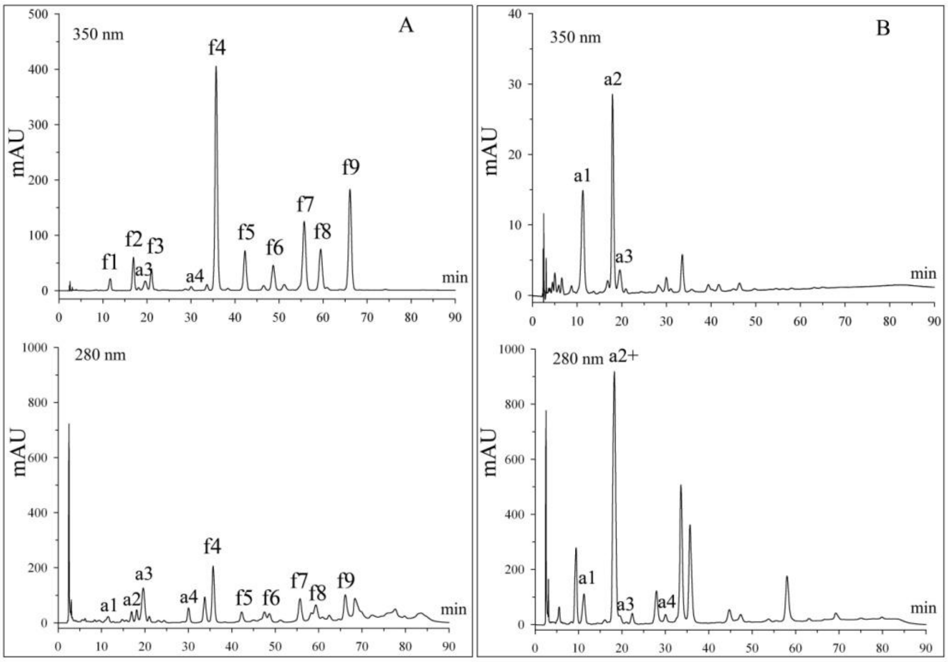


**Figure S2.** HPLC profiles of flower extracts from *Paeonia suffruticosa* cv. ‘Gunpohden’ (A) and *Paeonia tenuifolia* (B) at 350 nm and 280 nm. a1, Cy3G5G; a2, Cy3G; a3, Pn3G5G; a4, Pn3G; f1, Qu3G7G; f2, Km3G7G; f3, Is3G7G; f4, Lu7G; f5, Lu7Neo; f6, Ap7G; f7, Ap7Neo; f8, Ch7G; and f9, Ch7Neo. The unlabeled peaks are unknown peaks. Peak a2+ was a mixture that included a2 and an unknown compound.

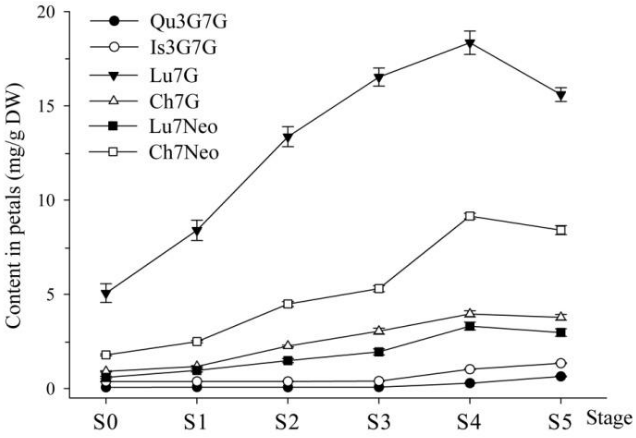


**Figure S3.** The accumulation of colorless flavonoids in *Paeonia suffruticosa* cv. ‘Gunpohden’ petals during flower development.

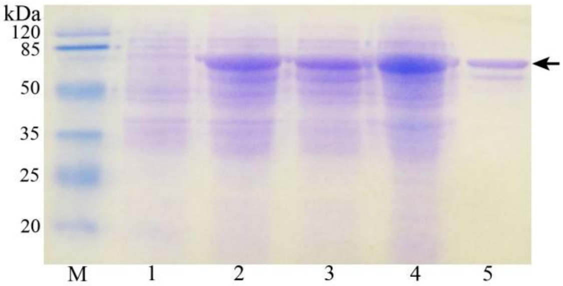


**Figure S4.** SDS-PAGE of recombinant PsAOMT. Lane M, protein size markers; Lanes 1–2, total *E. coli* protein before or after induction; Lanes 3–4, fragmented *E. coli* supernatant and pellet after induction with fusion protein; and Lane 5, purified MBP-PsAOMT fusion protein.

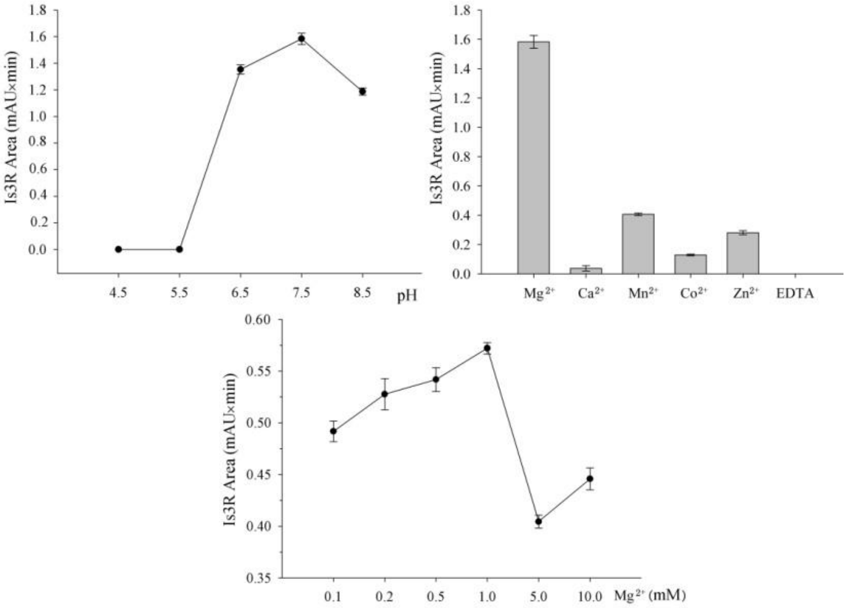


**Figure S5.** The catalytic activity of PsAOMT with quercetin 3-*O*-rutinoside (Qu3R) as a substrate under different reaction conditions, including a pH gradient of buffer solutions, the presence of different divalent cations, and various $Mg^{2+}$ concentrations. The mAU areas of the methylated product and isorhamnetin 3-*O*-rutinoside (Is3R) are presented as the means of triplicate experiments.

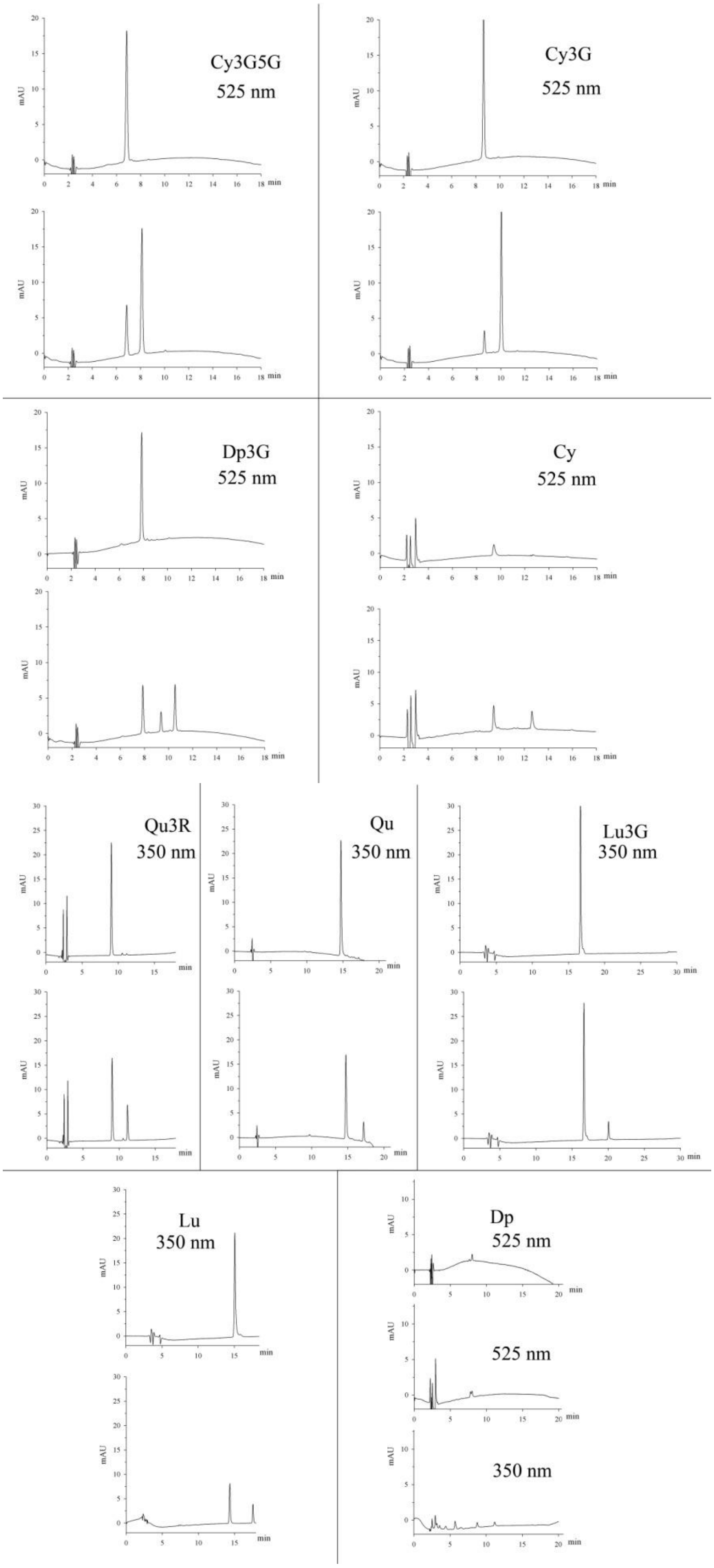


**Figure S6**. Profiles of products methylated by recombinant PsAOMT using a series of substrates in the *in vitro* system by HPLC analysis.

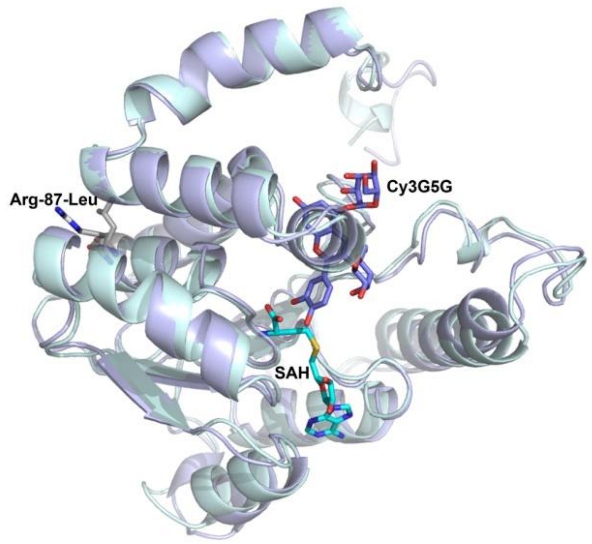


**Figure S7.** Superimposed 3D-models of PsAOMT and PtAOMT are represented as cartoons; PsAOMT and PtAOMT are labeled in light blue and pale cyan, respectively. SAH, cyanidin 3,5-di-*O*-glucoside (Cy3G5G), and the key amino acid residue Arg-87-Leu are represented as sticks and labeled in cyan, slate and white, respectively. The oxygen, nitrogen, and sulfur atoms are labeled, in red, blue, and yellow, respectively.

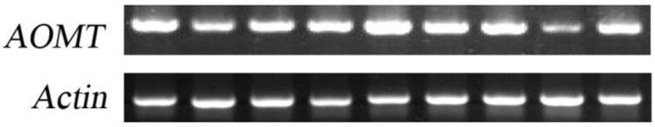


**Figure S8.** The expression of *AOMT*s in transgenic tobacco lines according to RT-PCR. Lanes 1–5, transgenic lines with 35S::*PsAOMT*; Lanes 6–9, transgenic lines with 35S::*PtAOMT*. The expression of *Actin* (GQ339768) was used as standard.

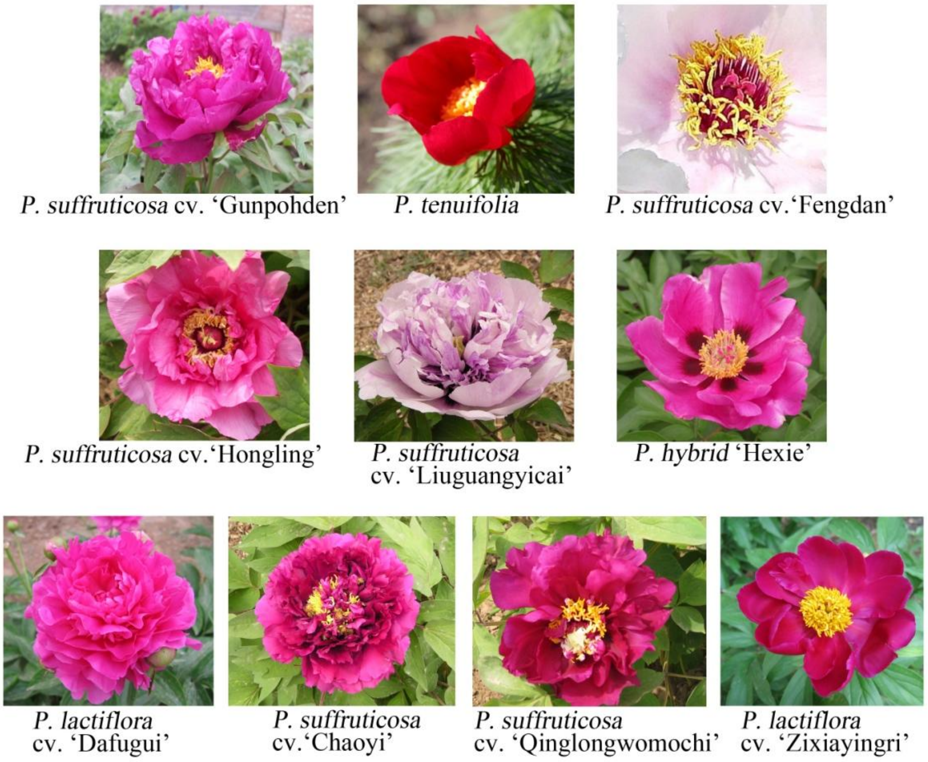


**Figure S9.** Flower phenotypes of different *Paeonia* cultivars used in this study.

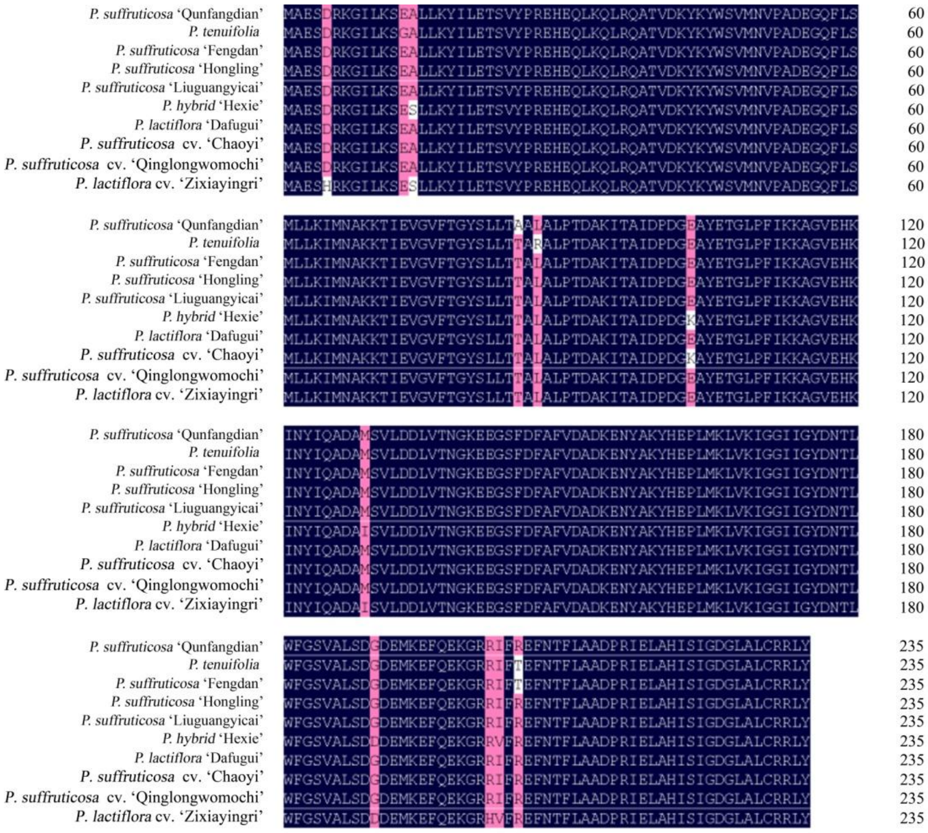


**Figure S10.** Amino acid sequence alignments of the AOMTs from ten *Paeonia* plants.

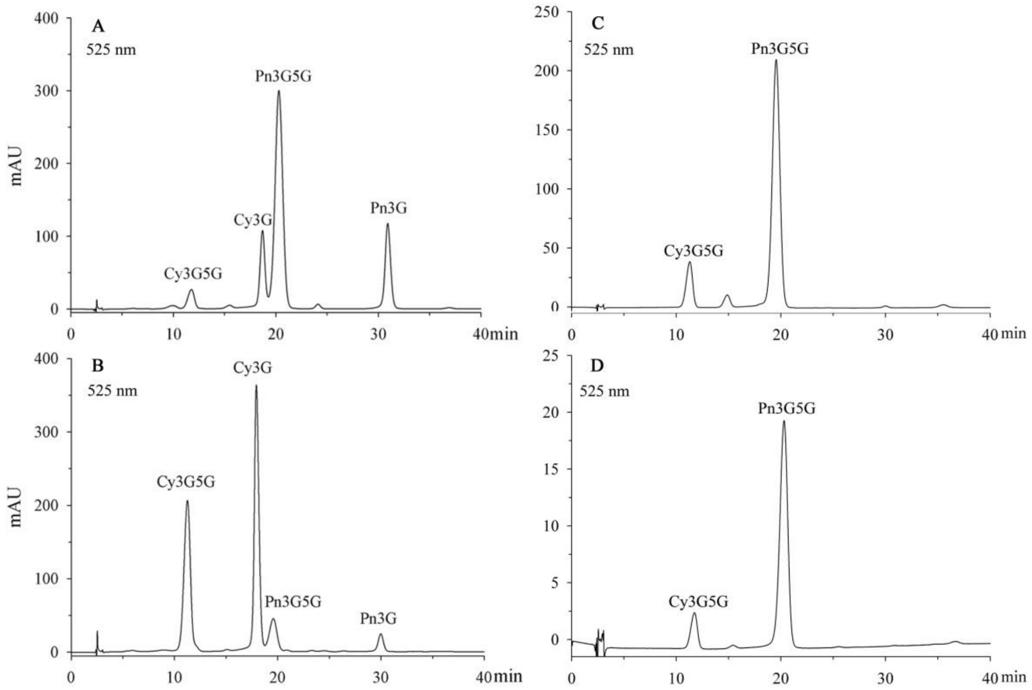


**Figure S11.** HPLC profiles of anthocyanins in flower petals. A, *Paeonia suffruticosa* cv. 'Gunpohden'; B, *Paeonia tenuifolia*; C, *Paeonia hybrid* ('Hexie'); D, *Paeonia lactiflora* cv. 'Dafugui.'

**Table S1** The primer sequences used in site-directed mutagenesis and qPCR.

| Primer Name | Oligonucleotide sequence |
|---|---|
| AOMT-forward | ATGGCCGAGTCAGATAGGAAGGGT |
| AOMT-reverse | TAAAATGATCCTTCTGCCATGCT |
| PtAOMT–G13E-forward | TATTCTTAAAAGTGAAGCCCTTTTG |
| PtAOMT–G13E-reverse | TCACTTTTAAGAATACCCTTCCTAT |
| PtAOMT–T85A-forward | TTATTCTCTTCTCACTGCCGCTCGT |
| PtAOMT–T85A-reverse | CAGTGAGAAGAGAATAACCAGTAAAG |
| PtAOMT–R87L-forward | TCACTACCGCTCTTGCTCTGCCTAC |
| PtAOMT–R87L-reverse | AGAGCGGTAGTGAGAAGAGAATAAC |
| PtAOMT–T205R-forward | GGCGCATCTTTAGAGAGTTCAACAC |
| PtAOMT–T205R-reverse | CTAAAGATGCGCCTGCCCTTTTCCTG |
| PsAOMT–L87R-forward | CTTCTCACTGCCGCTCGTGCTCTGC |
| PsAOMT–L87R-reverse | CGAGCGGCAGTGAGAAGAGAATAACC |
| PsAOMT–L87A-forward | TCTCACTGCCGCTGCTGCTCTGCCT |
| PsAOMT–L87A-reverse | GCAGCGGCAGTGAGAAGAGAATAACC |
| AOMT-qPCR-forword | TAAGAAGGCTGGAGTGGAGCATAAG |
| AOMT-qPCR- reverse | GGCATAGTTTTCCTTGTCAGCATCC |
| Actin-forward (For *Paeonia*) | GCAGTGTTCCCCAGTATT |
| Actin-reverse (For *Paeonia*) | TCTTTTCCATGTCATCCC |
| Actin-forward (For transgenic lines) | GCCAACAGAGAGAAAATGACCC |
| Actin-reverse (For transgenic lines) | TCATGGATGGCTGGAAGAGGACTTC |

**Table S2** A summary of ten specimens, including information regarding the flower color, chromatic parameters, total anthocyanin content, methylation levels of the anthocyanins, and the key amino acid(s) of the AOMTs.

| | *Paeonia suffruticosa* cv. 'Gunpohden' | *P. tenuifolia* | *P. suffruticosa* cv. 'Fengdan' (flower disc) | *P. suffruticosa* cv. 'Hongling' | *P. suffruticosa* cv. 'Liuguangyi cai' | *P. hybrid* 'Hexie' | *P. lactiflora* cv. 'Dafugui' | *P. suffruticosa* cv. 'Chaoyi' | *P. suffruticosa* cv. 'Qinglongwo mochi' | *P. lactiflora* cv. 'Zixiayingri' |
|---|---|---|---|---|---|---|---|---|---|---|
| Flower color | purple-red | vivid red | purple-red | purple-red | Purple-red | Purple-red | Purple-red | purple-red | purple-red | red-purple |
| RHSCC | 77B | 61A | — | 61C | 82C | 72B | 71D | 71B | 71A | 61A |
| *L** | 35.5 | 31.5 | 75.9 | 51.9 | 75.8 | 35.3 | 46.1 | 33.94 | 25.26 | 22.47 |
| *a** | 36.2 | 41.5 | -7.4 | 40.7 | -0.7 | 47.1 | 45.2 | 47.35 | 33.51 | 34.37 |
| *b** | -16.0 | 9.4 | 5.6 | -7.2 | 5.6 | -17.0 | -13.1 | -10.60 | -4.15 | 0.64 |
| *C** | 39.7 | 42.6 | 9.3 | 41.4 | 5.8 | 50.2 | 47.2 | 48.55 | 33.85 | 34.37 |
| Hue | -23.9 | -12.9 | -10.2 | -10.15 | -15.05 | -20.2 | -16.5 | -12.59 | -6.86 | 1.07 |
| Total anthocyanins (mg/g DW) | 10.73 | 13.25 | 2.32 | 13.05 | 2.54 | 7.64 | 4．86 | 27.62 | 45.25 | 15.93 |
| TF | 32.70 | 0.72 | — | 42.28 | 33.1 | 38.23 | 23.37 | 24.18 | 41.71 | 23.55 |
| CI | 3.05 | 0.05 | — | 3.24 | 13.06 | 5.28 | 4.81 | 0.88 | 0.92 | 1.48 |
| Methylation% | 94 | 24 | 95 | 91 | 86 | 87 | 89 | 55 | 67 | 68 |
| Amino acid at 87 position | Leucine | Arginine | Leucine | Leucine | Leucine | Leucine | Leucine | Leucine | Leucine | Leucine |

The data of chroma parameters (RHSCC, *L**, *a**, *b**, *C** and hue), TA, TF and CI were cited from Li CH (2010, The flavonoid composition in tree peony petals and their effects on the coloration, PhD dissertation) and Jia N (2008, Studies on petal coloration mechanism and chemotaxonomy of herbaceous peony, Master dissertation).

**Table S3** Identification of products methylated by recombinant PsAOMT using a series of substrates in the *in vitro* system.

| Substrate (µM) and reaction duration (min) | Wavelength nm | Retention time min | *m/z* positive mode | *m/z* negative mode | Identification |
|---|---|---|---|---|---|
| Cy3G5G 25 µM-10 min | 525 | 4.9 | 287.1(3.59),449.1(8.97), 611.1(100) | | Cyanidin 3,5-di-*O*-glucoside |
| | | 6.2 | 301.0(3.39), 625.1(100) | | Peonidin 3,5-di-*O*-glucoside |
| Cy3G 25 µM-10 min | 525 | 6.4 | 287.0(69.94), 449.1(54.29) | | Cyanidin 3-*O*-glucoside |
| | | 7.8 | 301.1(98.70), 463.1(100) | | Peonidin 3-*O*-glucoside |
| Dp3G 25 µM-10 min | 525 | 6.1 | 303.1(100), 465.1(75.44) | | Delphinidin 3-*O*-glucoside |
| | | 7.6 | 317.0(98.05), 479.1(100) | | Petunidin 3-*O*-glucoside |
| | | 8.9 | 331(74.91), 493.1(100) | | Malvidin 3-*O*-glucoside |
| Qu3R 25 µM-10 min | 350 | 9.8 | 303.0(100), 465.1(35.01), 611.1(28.62) | | Quercetin 3-*O*-rutinoside |
| | | 11.9 | 317.1(100), 479.0(30.55), 625.1(22.67) | | Isorhamnetin 3-rutinoside |
| Qu 25 µM-10 min | 350 | 14.6 | 303.0(100) | 300.9(100) | Quercetin |
| | | 18.1 | 149.0(100), 317.1(33.64) | 311.0(100) | Isorhamnetin |
| Lu7G 25 µM-10 min | 350 | 10.2 | 287.0(100), 449.1(49.36) | | Luteolin 7-*O*-glucoside |
| | | 12.4 | 301.1(100), 463.1(46.08) | | Chrysoderol 7-*O*-glucoside |
| Lu 25 µM-10 min | 350 | 15.1 | 287.0(100) | | Luteolin |
| | | 18.2 | 301.0(100) | | Chrysoderol |
| Cy 50 µM-10 min | 525 | 11.4 | 287.0(100) | | Cyanidin |
| | | | 301.2(31.10), 287.0(20.42), 276.0(72.62), 149.0(100) | | Peonidin |
| Dp 50 µM-10 min | 350 | 8.1 | 393.0(100),149.0(79.49), 132.0(75.05), 471.8(23.54) | 468.9(100), 409.0(26.44),376.9(22.32),276.8(14.04) | Unknown |
| | | 11.4 | 132.0(100), 276.1(85.16), 395.0(76.54), 471.9(52.31) | 392.8(84.76), 468.8(100) | Unknown |
| | | 13.8 | 132.0(100),149.0(81.39), 276.0(37.42), 309.7(89.57), 319.0(42.72) | 297.0(100), 316.9(99.49) | Unknown |

**Table S4** PtAOMT-R87L activities with major substrates under optimized assay conditions.

| Substrates | PtAOMT-R87L<br>$K_m$<br>*μM* | <br>$V_{max}$<br>*nM s*$^{-1}$ | <br>$K_{cat} \times 10^{-3}$<br>*s*$^{-1}$ | <br>$K_{cat}/K_m$<br>*M*$^{-1}$*s*$^{-1}$ | <br>Specific Activity<br>*pkat mg*$^{-1}$ |
|---|---|---|---|---|---|
| Cyanidin 3,5-di-*O*-glucoside | 8.51 (1.39) | 14.94 (0.73) | 106.71 (5.21) | 13010 (1428) | 1494 (73) |
| Cyanidin 3-*O*-glucoside | 3.53 (0.31) | 17.59 (0.30) | 125.66 (2.16) | 36058 (3045) | 1759 (30) |
| Delphinidin 3-*O*-glucoside | 27.27 (1.49) | 19.06 (0.88) | 0.14 (0.01) | 4999 (82) | 1906 (88) |
| Quercetin 3-*O*-rutinoside | 8.09 (0.25) | 9.31 (0.97) | 0.07 (0.01) | 8253 (923) | 931 (97) |
| Luteolin 7-*O*-glucoside | 48.88 (2.74) | 1.26 (0.24) | 0.01 (0.00) | 189 (47) | 126 (24) |

Data are expressed as means (±SE) of triplicate assays.

**Table S5** Anthocyanins in strawberry fruits as detected by HPLC-MS (demonstrated in Figure 6).

| Peak No. | Compound | Peak No. | Compound |
|---|---|---|---|
| a1 | Pg 3,5-di-*O*-glucoside | a4 | Cy 3-*O*-malonylglucoside |
| a2 | Pg 3-*O*-glucoside | a5 | Pg 3-*O*-malonylglucoside |
| a3 | Pg 3-*O*-rutinoside | a6 | Pn 3-*O*-malonylglucoside |
